# Exploring the Enhancement of Exchange Bias in Innovative Core/Shell/Shell Structures: Synthesis and Magnetic Properties of Co-Oxide/Co and Co-Oxide/Co/Co-Oxide Inverted Nanostructures

**DOI:** 10.3390/nano13050880

**Published:** 2023-02-26

**Authors:** Maral Ghoshani, Morteza Mozaffari, Mehmet Acet, Mahshid Hosseini, Daryoosh Vashaee

**Affiliations:** 1 Department of Physics, Faculty of Physics, University of Isfahan, Isfahan 81746-73441, Iran; 2 Instituto Regional de Investigación Científica Aplicada (IRICA) and Departamento de Física Aplicada, Universidad de Castilla-La Mancha, 13071 Ciudad Real, Spain; 3 Faculty of Physics and CENIDE, Universität Duisburg-Essen, 47048 Duisburg, Germany; 4 Physics Department, North Carolina State University, Raleigh, NC 27606, USA; 5 Department of Materials Science and Engineering, North Carolina State University, Raleigh, NC 27606, USA; 6 Department of Electrical and Computer Engineering, North Carolina State University, Raleigh, NC 27606, USA

**Keywords:** nanostructures, magnetic material, exchange bias systems, core/shell, inverted core/shell, Co-oxide/Co

## Abstract

In this study, we investigate the enhancement of exchange bias in core/shell/shell structures by synthesizing single inverted core/shell (Co-oxide/Co) and core/shell/shell (Co-oxide/Co/Co-oxide) nanostructures through a two-step reduction and oxidation method. We evaluate the magnetic properties of the structures and study the effect of shell thickness on the exchange bias by synthesizing various shell thicknesses of Co-oxide/Co/Co-oxide nanostructures. The extra exchange coupling formed at the shell–shell interface in the core/shell/shell structure leads to a remarkable increase in the coercivity and the strength of the exchange bias by three and four orders, respectively. The strongest exchange bias is achieved for the sample comprising the thinnest outer Co-oxide shell. Despite the general declining trend of the exchange bias with Co-oxide shell thickness, we also observe a nonmonotonic behavior in which the exchange bias oscillates slightly as the shell thickness increases. This phenomenon is ascribed to the dependence of the antiferromagnetic outer shell thickness variation at the expense of the simultaneous opposite variation in the ferromagnetic inner shell.

## 1. Introduction

The exchange-bias phenomenon was reported for the first time by Meiklejohn and Bean in fine Co particles coated with a thin antiferromagnetic (AFM) cobalt-oxide layer having a Néel-temperature *T_N_ = 293* K [1,2,3]. The exchange bias in this system is mainly characterized by a horizontal shift in the hysteresis loop when measured after cooling the sample through *T_N_* under a magnetic field [1,2,3]. The magnetic moments of the AFM phase adjacent to the ferromagnetic (FM) border acquire an uncompensated magnetic moment parallel to that of the FM component already saturated by the field. This alignment introduces a new unidirectional anisotropy in the direction of the cooling field, thereby causing a horizontal shift in the hysteresis loop [1,2,3]. Exchange bias has been mainly discussed using the FM/AFM interface pinning picture for thin films, composites, and core/shell systems. FM/AFM structures are potentially useful for controlling magnetic properties [1,4,5] and the development of permanent magnets [3,6,7], spin-valve structures [8,9,10], spincaloritronic materials [11,12,13], thermoelectric materials [14,15,16], quantum materials [17], and overcoming the superparamagnetic limit for high-density recording media [18,19,20]. Exchange bias has been studied in other nanostructured materials having a combination of differently ordered magnetic components, including ferrimagnetic/AFM and FM/ferrimagnetic [3,21,22,23,24,25]. Most of the research on exchange-bias systems in transition-metal cores and corresponding oxide layers was performed by chemical methods. These methods result in poor development of the AFM shell [2,3,26,27,28,29,30], leading to a low-blocking, exchange bias temperature. The effects of different parameters, such as interfacial roughness, particle size, magnetic and nonmagnetic defects, and the shape of nanoparticles in conventional core/shell structures, have been investigated [3]. The core/shell with a metal shell has rarely been studied [31,32,33,34,35]. Soares et al. synthesized the CoFe_2_O_4_/CoFe_2_ by reducing CoFe_2_O_4_ in the hydrogen to find out the critical shell thickness in which the core couples to the shell through the exchange-spring mechanism. Moreover, they studied exchange bias in this nanostructure [33,35]. Soares et al. also claimed that the FeO phase in the CoFe_2_O_4_/CoFe-FeO nanoparticle affects the magnetic properties [34]. Zhou et al. studied the effect of field cooling on the exchange bias of CoO/Co nanoparticles. They showed that the first exchange bias increased as the cooling field was increased, and second by increasing the size of the CoO particles with the increase in CoO content, the exchange bias decreased [31]. Recently, there has been a growing interest in new exchange-bias systems known as inverted structures in which the core is AFM, and the shell is a ferro/ferrimagnet material [26,27,29,36,37,38,39,40,41,42]. The AFM structure, which can be better controlled in the core than in the shell, is employed to overcome several limitations of conventional systems [26,27,40]. Depending on the magnetic transition temperatures of core/shell structures, the inverted structures are referred to as “single inverted” and “doubly inverted” core/shell [27,29,36,40,43]. Single inverted systems have a ferro/ferrimagnet with a higher Curie temperature *T_C_* than *T_N_*. In contrast, doubly inverted systems have a *T_C_* smaller than *T_N_* [27,40]. The properties of single inverted structures, such as FeO/Fe_3_O_4_, CoO/Fe_3_O_4_, CoO/γ-Fe_2_O_3_, CoO/CoFe_2_O_4_, and Cr_2_O_3_/Fe_3_O_4_ have been studied experimentally and theoretically [26,36,38,39,44,45,46]. Kavich et al. studied the exchange bias in single inverted FeO/Fe_3_O_4_ nanoparticles. They showed that the exchange-bias blocking temperature is almost constant for different magnetic fields ranging from 2 to 16 kA/m arising from exchange coupling between the core and the shell [36]. Winkler et al. compared two core/shell structures with a nonmagnetic core and an AFM core covered with a ferrimagnetic shell. They showed that the major contribution to the large coercivity in CoO/CoFe_2_O_4_ is related to the exchange coupling between the AFM core and the ferrimagnetic shell [38]. Liu et al. revealed a large exchange bias with significant thermostability in the singly inverted CoO/γ-Fe_2_O_3_ core structure. Moreover, they tuned the exchange bias by varying the temperature and the size of the cooling field, showing that the uncompensated spins at the interface between the core and shell play a key role in the observed exchange bias [26]. Research on the singly inverted core/shell indicates that the exchange bias strengthens with increasing AFM core size, while it weakens when the thickness of the FM shell increases [26,36,44]. On the contrary, the doubly inverted structure shows different features, such as the nonmonotonic variation in coercivity and exchange bias with variations in AFM core diameter and ferrimagnetic shell thickness [27,29,40,43]. Salazar-Alvarez et al. studied the magnetic properties of MnO/Mn_3_O_4_ core/shell particles that were synthesized chemically. They showed that the coercivity and exchange bias changed nonmonotonically by increasing the core diameter. These behaviors have been ascribed to the variations in the building of the AFM layers and their influence on the uncompensated spins at the core–shell interface [40]. Vasilakaki et al. studied doubly inverted core/shell structures with Metropolis Monte Carlo simulations. They showed that the dependencies on the thickness of the FM shell in small and large particles have an opposite trend [27]. However, only a few investigations assess exchange bias in inverted core/shell/shell structures [47,48,49]. Sort et al. studied exchange bias in AFM/FM/AFM thin films. They showed that coercivity and exchange bias are larger in AFM/FM/AFM structures than in AFM/FM and FM/AFM systems and that the influence of having two AFM/FM interfaces is the summation of each interface’s contribution [5]. Various magnetic properties and exchange-bias effects in conventional Co/Co-oxide structures have been investigated. However, the inverted cobalt oxide/cobalt structure has been somewhat overlooked.

In this work, we prepare core/shell Co-oxide/Co and core/shell/shell Co-oxide/Co/Co-oxide nanostructures with reduction and oxidation methods to assess the influence of having two AFM/FM interfaces in the inverted structure. Moreover, nanostructures with various shell thicknesses are synthesized by controlling the synthesis process parameters to study the effect of shell thickness on magnetic properties, such as coercivity and exchange bias. The results indicate that increasing the thickness of the AFM phase results in a nonmonotonic change in the exchange bias, contrary to conventional core/shell structures. We correlate the exchange bias variation to the number of AFM domains and the change in uncompensated spins at the interface.

## 2. Experimental Methods

### 2.1. Synthesis of Samples

The raw material Co_2_O_3,_ with a minimum purity of 99%, was purchased from Merck Company (Darmstadt, Germany).

To synthesize CoO, Co_2_O_3_ has been reduced in the extra pure hydrogen gas in a controlled atmosphere in the electrical tube-furnace operated between 180 °C and 500 °C for 1 h to choose the best temperature that results in “almost” pure CoO nanoparticles. The achieved product (CoO identified by XRD) is then used to synthesize Co-oxide/Co inverted core/shell nanostructures by reducing the extra pure hydrogen at 370 °C, utilizing the same furnace. The amount of hydrogen required to reduce 80% of Co-oxide is calculated based on the weight of the Co-oxide powder and estimated the particle and the crystallite sizes of the powder. Lastly, the obtained Co-oxide/Co nanostructures (Co-oxide /Co identified by XRD) are oxidized in the pure oxygen at 260 °C for different time intervals to obtain Co-oxide /Co/ Co-oxide core/shell/shell nanostructures with various shell thicknesses. All nanoparticles collected under the name OMO-series and are identified with their oxidation times: OMO(0), OMO(5), OMO(20), OMO(40), OMO(60), and OMO(80).

### 2.2. Structural and Magnetic Characterization Methods

The X-ray diffraction (XRD) patterns of all samples were recorded by a Philips X’Pert Pro diffractometer (Košice, Slovak Republic)operating in reflection mode with Cu Kα radiation (λ=1.5406 Å) at room temperature and the scanning rates of 0.04%. XRD patterns are identified using Panalytical HighScore software. The mean crystallite sizes of the samples are estimated via Scherrer’s formula:

$$d=\frac{0.9\lambda }{B\cos\;\theta }$$

where d is the mean crystallite size, θ is the Bragg angle of the most intense peak of each phase, λ is the X-ray wavelength, and B is full width at half maximum (FWHM) in radians. The quantitative analysis of the XRD data is conducted with a full pattern-fitting procedure based on the Rietveld method using the MAUD program. Magnetic measurements are performed using a Quantum Design MPMS XL SQUID Magnetometer(Quantum Design North America, San Diego, CA, USA). Hysteresis loops are obtained at 5 K following sample cooling from room temperature in a 4000 kA/m magnetic field.

The morphology and microstructures of the samples were studied using a Field-emission transmission electron microscope (Talos F200X G2) (Thermo Fisher Scientific, Waltham, MA, USA). 

## 3. Study Results and Discussion

### 3.1. Structural Charactrization

**Figure 1:**
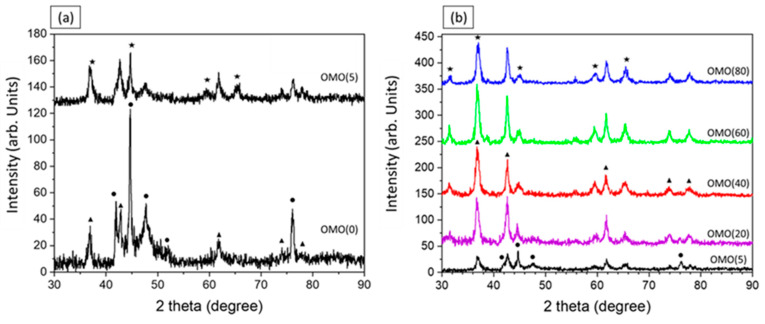
XRD patterns of the OMO-series (**a**) OMO(0) and OMO(5) and (**b**) in various oxidation times; the symbols next to different reflections indicate their assigned phase: (●) Co (00-001-1254 PDF card No.), (▲) CoO (00-043-1004 PDF card No.), and (★) Co_3_O_4_ (01-080-1533 PDF card No.).

**Figure 2:**
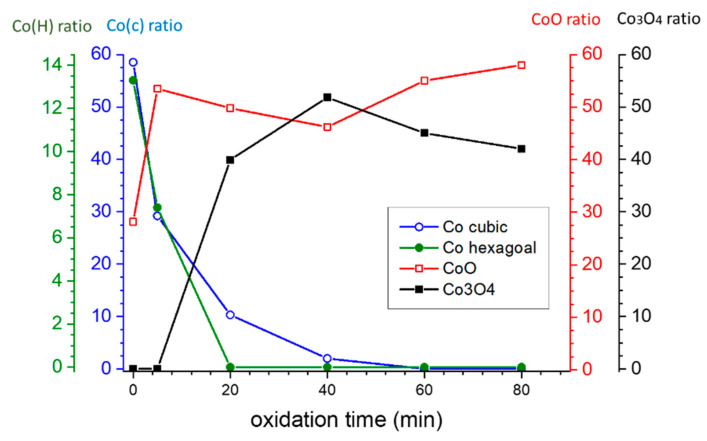
Evolution of the Phase Ratio with Respect to Oxidation Time in the OMO-Series. The connecting lines are to aid visual tracking of the phase variations.

Figure 1 demonstrates the XRD patterns of the OMO-series samples. The name of each sample is labeled, and the corresponding phases are marked for the pattern. The XRD data of OMO(0) and OMO(5) are separately shown in Figure 1a to reveal differences in the intensities of the peaks after the first oxidation process. It can be seen that the prominent peaks on the XRD pattern of OMO(0) are related to the Co-phases, while several lower-intensity peaks of the f.c.c CoO-phase is also visible. On the XRD pattern of OMO(5), despite the presence of Co- and CoO-phases, some peaks are related to the Co_3_O_4_ phase. As expected, on oxidation for 5 min, the intensity of the Co-oxide peaks (firstly from CoO and eventually from Co_3_O_4_) increase at the expense of the metallic Co peaks. The evolution of the volume percentage of Co-oxides and Co phases, represented in Table 1, was estimated from the pattern fits utilizing the MAUD program. It is seen that in OMO(0), 70% of the Co-oxide core is reduced to Co. By extending the oxidation time in the core/shell/shell structure (Figure 1b), the intensities of the Co-phase peaks decrease. Samples oxidized for longer than 20 min show a significant presence of Co_3_O_4_, with some CoO remaining but no metallic Co. The prolonged oxidation at high temperatures causes a concomitant crystallization process, as reflected by the relatively sharp peaks in the XRD patterns. The evolution of the phase ratio with respect to oxidation time is shown in Figure 2, which allows for the estimation of the shell and core thickness based on the volume percentage of the phases. The variation in the Co-oxide ratio versus oxidation time indicates that while there is an overall upward trend, the largest increase takes place within the first 20 min. Additionally, the continuous phase transition of cobalt oxide from CoO to Co_3_O_4_ during oxidation has been taken into account in the study of the magnetic properties.

**Figure 3:**
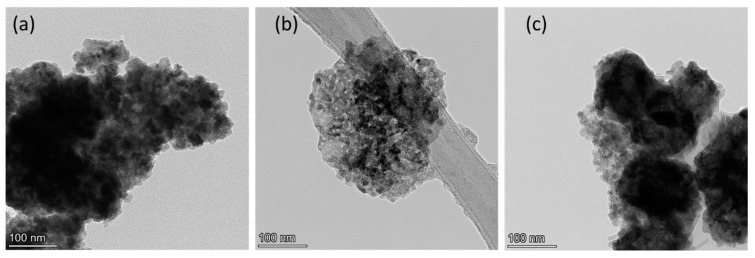
TEM micrographs of Co oxide/Co/Co Oxide nanoparticles oxidized for (**a**) 0, (**b**) 5, and (**c**) 40 min.

Figure 3 shows the TEM micrographs of the OMO(0), OMO(5), and OMO(40) samples. They show the presence of rough, spherical aggregated particles. Nanoparticles aggregate due to surface particle interactions, high synthesis temperature, and high magnetization. A longer oxidation time in the temperature does not have much effect on the particle size.

**Figure 4:**
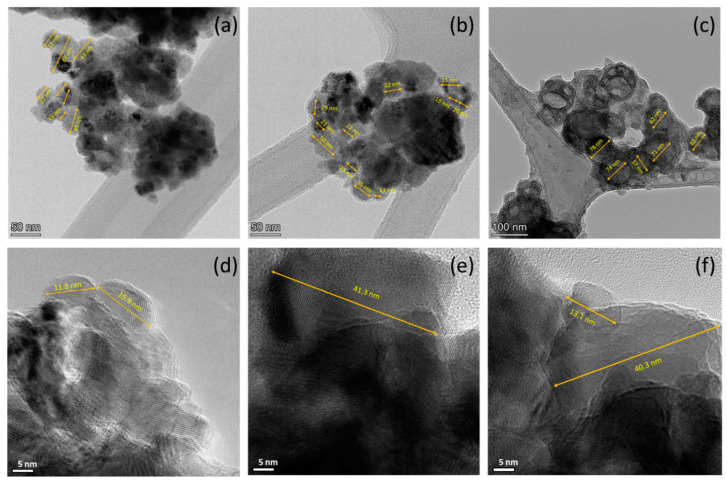
High-magnification TEM micrographs of (**a**,**b**) OMO(0), (**c**) OMO(5), and (**d**–**f**) OMO(40) corresponding to different resolutions. The sizes of some selected nanoparticles are marked on the image.

Figure 4 shows TEM results of OMO(0), OMO(5), and OMO(40) at a higher resolution, so nanoscale grains with specified dimensions can be seen. These small grains are in the 20 to 70 nm range. The longer thermal treatments produce more aggregation/sintering making it difficult to find small separated particles. However, more prolonged oxidation does not significantly affect the particle size. 

**Figure 5:**
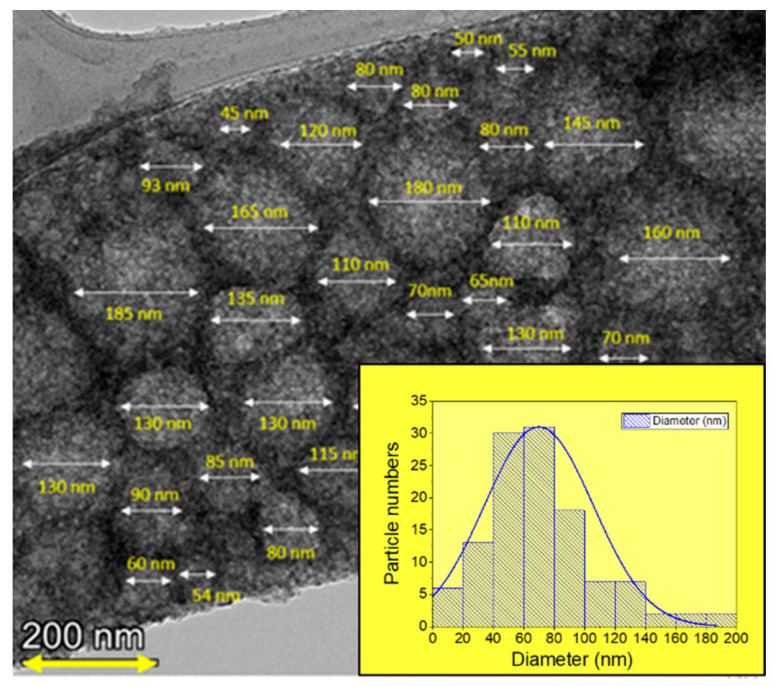
TEM micrograph of OMO(40) and the particle-size histogram.

Figure 5 shows a TEM micrograph of OMO(40) and is one of the images used to measure the size distribution. The nanoparticles are found to be approximately spherical and agglomerated to each other tightly. The particle size histogram shows a wide distribution and an average diameter of 70 nm.

**Figure 6:**
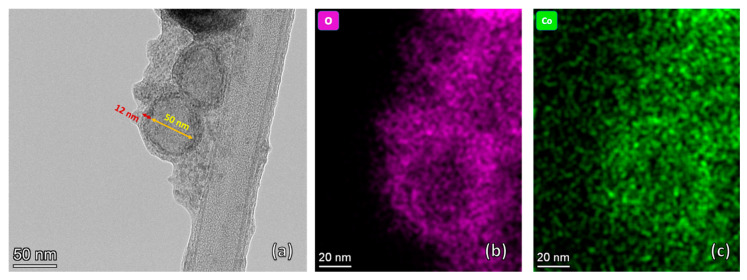
TEM image (**a**) and EDS mapping images (**b**,**c**) of OMO(5) showing a core/shell structure with oxygen (**b**) and cobalt (**c**).

Figure 6 illustrates the TEM and the EDS mapping images of OMO(5) samples. In the TEM image, a spherical core/shell nanoparticle is observed with a size of 62 nm. As can be seen, the darker outer ring with a thickness of 12 nm and the lighter inner part with a diameter of 50 nm are the shell and the core, respectively. EDS mapping is employed for the elemental analysis. In Figure 6b,c, the purple color indicates the presence of oxygen, with its brighter part representing less oxygen concentration. The green color is related to the Co phases comprising a more uniform distribution, although the color becomes lighter, moving from the center of the particle to the sides. These results confirm that these nanoparticles are core/shells, with the outermost layer being cobalt oxide.

**Figure 7:**
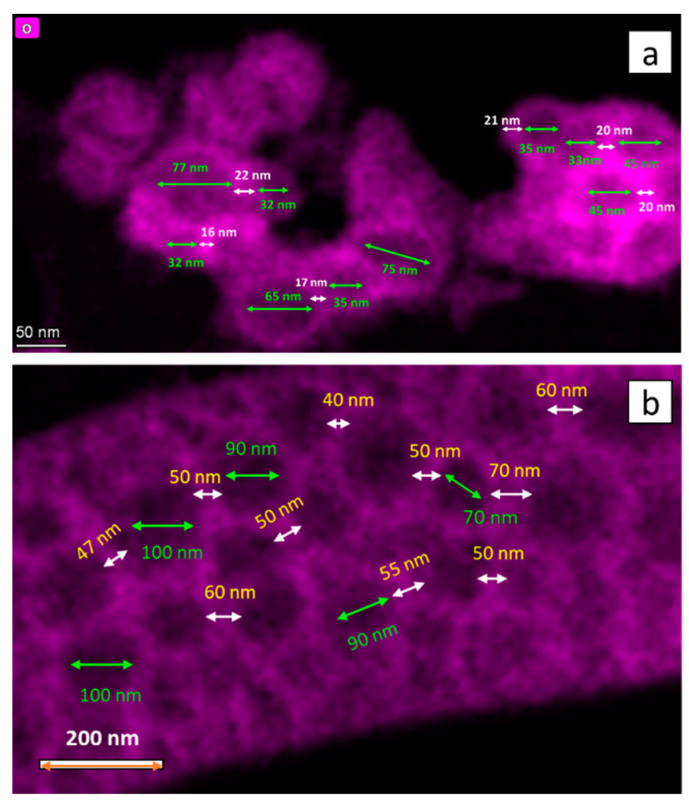
EDS mapped images of core/shell nanostructures. (**a**) shows OMO(5) and (**b**) illustrates OMO(40), displaying the presence of a Co oxide shell in the structure The core size is indicated by green arrows, while the shell thickness is indicated by white arrows.

Figure 7a,b show EDS mapping images of OMO(5) and OMO(40), respectively. From these images, it is possible to estimate the variation in core and shell sizes. There is a broad distribution in the core size, with the average estimated to be around 70 nm. The average shell thickness of OMO(5) and OMO(40) are around 10 and 26 nm, respectively. These dimensions show that the outer CoO shell thickness increases by increasing the oxidation time.

**Figure 8:**
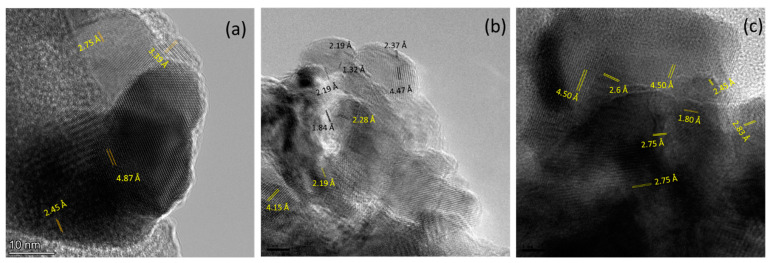
High-Resolution TEM Images of (**a**) OMO(5) and (**b**,**c**) OMO(40) showing the presence of CoO, Co_3_O_4,_ and Co phases.

The HR images in Figure 8 show various crystalline domains, exhibiting contrasts consistently with a mixture of metallic Co and Co-oxides phases. The observed interplanar spacings 2.37, 2.75, and 4.47 Å are consistent with the spacings of the fcc Co_3_O_4_ (111), (220), and (222) planes, respectively, whereas 2.45 Å is consistent with the spacing of the fcc CoO (111) plane. Moreover, 2.19 Å and 1.84 Å are consistent with the spacings of the hexagonal Co (100) and (101) planes, respectively. These results indicate the coexistence of the three different phases and their interfaces. Moreover, nanoparticles exhibit good crystallinity.

### 3.2. Magnetic Characterization

**Figure 9:**
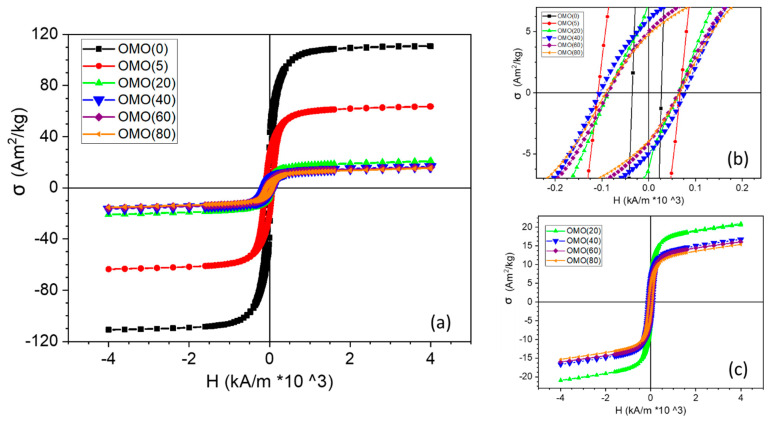
Hysteresis loops of core/shell/shell nanostructures at 5 K after field-cooling (4000 kA/m). (**a**) shows the overall loops, (**b**) is a close-up of the low-field portion of the loops, and (**c**) illustrates the effect of increasing oxidation time on the loops. (Oxidation times in minutes are indicated in the legend).

**Figure 10:**
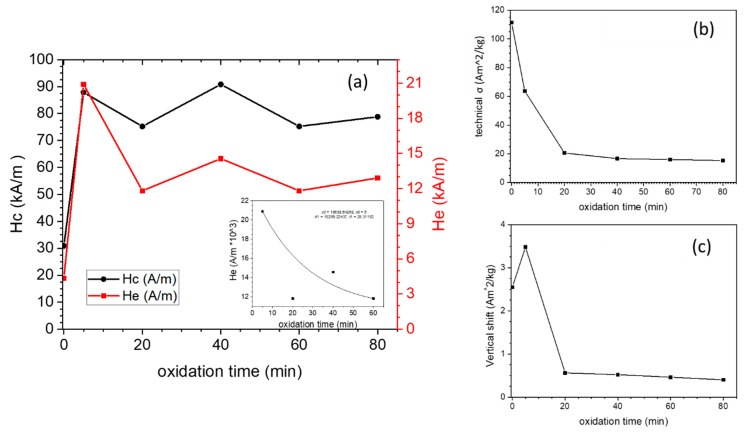
The effect of oxidation time on the magnetic properties of OMO-series. (**a**) Variation in the coercivity (H_c_) and exchange bias (H_e_), (**b**) variation in the technical saturation magnetization σ, and (**c**) variation in the vertical shift. The inset shows the fitted curve of the decrease in exchange bias with increasing oxidation time.

The field-cooled (4000 kA/m) hysteresis loops of Co-oxide/Co samples obtained by oxidation at various times, both on the full panels and their low field parts measured at 5 K, are shown in Figure 9. The technical saturation magnetization (@ 4000 kA/m) and coercivity change by increasing the oxidation time. All loops have horizontal and vertical shifts. The variations in magnetic parameters of all samples with respect to oxidation time are shown in Figure 10.

Figure 10b shows that the technical saturation magnetization, σ, is reduced upon oxidation, evidencing the progressive growth of a Co-oxide outer shell at the expense of the Co inner shell.

This is in good agreement with the results obtained from the XRD patterns. The OMO(0) sample has the largest saturation magnetization (111.4 Am^2^/kg) due to a thick Co shell. Since OMO(0) was oxidized for only 5 min, the saturation magnetization decreased to 63.7 Am^2^/kg, indicating that a large part of the cobalt shell had been oxidized. In the second stage of the 20 min oxidation, the magnetization again declines significantly to 20.63 Am^2^/kg. Following the increase in oxidation time, the decreasing trend continues (Figure 1c), but the variations are minimal and show that the outer oxide shell acts as a buffer layer. The normalization of the measured σ by the bulk magnetization of cobalt (σ_bulk_ = 160 Am^2^/kg) gives an estimation of the Co and Co-oxide weight fractions for each sample. The values are given in the first two columns of Table 1. Although they compare reasonably well with the Co and Co-oxide fractions obtained from the XRD data, the values obtained by magnetometry give systematically higher Co-fractions. This could be attributed to an FM-like contribution from the AF Co-oxide phase, mainly due to crystalline disorder or size effects, as reported before for other AF nanostructures [50,51,52,53,54,55]. Moreover, compared to XRD, magnetometry is much more accurate in detecting the presence of ferromagnetic phases. Based on this comparison, it can be concluded that the OMO(40), OMO(60), and OMO(80) contain a cobalt phase that was not detected by XRD. The thickness of the inner cobalt shell decreases with increasing oxidation times, which can be estimated from these results. These findings confirm that the OMO(0) has a core/shell structure, while the remaining samples have a core/shell/shell structure. The thickness of the inner cobalt shell can be estimated by the increasing oxidation times.

Figure 9b shows that the hysteresis loop is narrow for OMO(0) (Co-oxide/Co), while for the oxidized samples (Co-oxide/Co/Co-oxide), the hysteresis loops expand considerably. The coercivity of OMO(0) is around 32 kA/m, and after 5 min of oxidation (OMO(5)), it increases to 87.9 kA/m. Figure 10a indicates that the variation in coercivity with respect to the oxidation time is not uniform in the OMO-series and has a maximum for 40 min oxidation (90.9 kA/m) and a minimum for 20 and 60 min oxidation times (75.3 kA/m). The range of coercivity values reported in the literature on Co/Co-oxide nanoparticles varies from 16 to 800 kA/m [31,32,56,57,58,59,60,61,62]. It was shown that the coercivity is highly influenced by the particle size, domain structure, shape of the particle, and the thickness of each layer [31,32,42,56,57,62,63]. Srikala et al. reported a range of coercivities from 16 to 56 kA/m for Co/CoO nanoparticles with different sizes, ranging from 14 to 44 nm and with spherical and cubic morphologies [57]. Lopez et al. studied the reduction in CoO under high-vacuum annealing. They showed that at a high temperature, most portion of the CoO phase was reduced to metallic Co, resulting in the aggregation of smaller particles leading to the formation of larger particles. This led to an increase in saturation magnetization and a decrease in coercivity [32]. Compared to the reported results, OMO(0) would have multi-domain structured nanoparticles in multiple orientations because of its large particle size and proportion of the Co phase [56], leading to low coercivity. The results also showed that the extra AFM/FM interface leads to a significant increase in coercivity. In the core/shell/shell structures, the coercivity does not show a monotonic increase/decrease by increasing the oxidation time, although the fluctuation is small. It is known that coercivity enhancement significantly affects the exchange coupling in bimagnetic systems since the exchange coupling between the two layers inserts a new term in the anisotropy energy [1,2,3]. The variation in the thickness of the layer could affect the exchange coupling between layers.

On the one hand, the core/shell structure with a thin Co-oxide shell with low AFM anisotropy causes the FM spin to drag the AFM spins during their rotation when the magnetic field is reversed. This extra energy required to rotate the FM and AFM spins together increases the coercivity. For the thick Co-oxide layer, the anisotropy energy of the AFM Co-oxide phase is strong enough to pin the FM spin, thereby decreasing the coercivity while the exchange bias increases. Hence, the variation in the Co-oxide thickness affects the coercivity. On the other hand, the coercivity strongly depends on the average FM particle size [2,64,65]. For particles greater than the critical single domain size, the coercivity is inversely proportional to the diameter (d) Hc∝1d [65]. Therefore, by decreasing the particle size, the coercivity increases. The coercivity decreases with decreasing particle size for nanoparticles smaller than the critical single domain size. This means the coercivity is maximum at a critical single domain size [65]. Furthermore, the coercivity reduction may be due to the size decrease by the oxidation of a fraction of multi-domain cores, which is plausible given the relatively large size of the cores in this series. Additionally, any changes in the compaction of the nanostructures may also affect the magnetic properties due to strong variations in the magnetic interactions [28,66,67,68,69]. Gangopadhyay et al. studied the effects of interparticle interactions on magnetic properties. They showed that the influence of packing in all samples causes a decrease in the coercivity except for small Co nanoparticles. Moreover, they mentioned that exchange coupling in the core/shell structures contributes more than interparticle dipolar interaction on the overall magnetic properties [67]. In addition to the arguments given above, in general, the particle size distribution and the surface quality also affect the coercivity.

Figure 10a shows the amount of H_E_ (exchange-bias field), indicating the presence of exchange coupling between the CoO-core and the Co shell, Co-oxide shell, and Co shell. The values of the exchange-bias field extracted from the loops are 5, 20.9, 11.8, 14.6, 11.8, and 12.9 kA/m for the samples OMO(0), OMO(5), OMO(20), OMO(40), OMO(60), and OMO(80), respectively. The extra Co-oxide shell causes an increase in the exchange bias. Compared to the range of exchange bias values reported in the literature on Co/Co-oxide nanoparticles, the value achieved here is small [10,58,59,62,70]. Moreover, the reported exchange biases in the Co-oxide/Co nanostructure with 6 and 4 nm sizes are 64 and 200 kA/m, respectively [31,32]. Lopez Anton et al. claimed that the increase in the amount of Co phase due to the reduction process yields drastic variations in exchange bias [32]. Hence, these findings are in accordance with a large amount of Co in the Co-oxide/Co nanostructures. In the conventional thin film systems, it was established that $He\propto 1/t_{FM}$ and $He\propto t_{AFM}$ [1,2,3]. Indeed, corresponding relationships in core–shell and composite structures are more complicated than in thin film systems [3,71,72,73]. López-Ortega et al. have reported that for core/shell structures with an FM core, the exchange-bias field is proportional to $He\;\propto 1/d_{FM}$ [3,64]. Liu et al., studying the exchange bias in the inverted core/shell of CoO/γ-Fe_2_O_3_, revealed that the dependence of the exchange bias on the thickness of the ferrimagnet shell and AFM core is similar to the conventional structures [26]. On the other hand, in a doubly inverted core/shell, the variation in the exchange bias versus the thickness of the ferrimagnet shell and AFM core is nonmonotonic [27,29,40,74]. Moreover, as the uncompensated spins contribute to the exchange bias, the number of available uncompensated spins significantly decreases as the size of particles increases [40,75,76,77,78]. Therefore, the size of the particles has a significant effect on the exchange bias. Thus, apart from the relation between the exchange bias and Co per Co-oxide ratio, small and nearly spherical nanoparticles agglomerate due to the high magnetization of cobalt, making several larger particles with low exchange bias. Interestingly, the exchange bias nearly quadrupled due to a two-fold AFM/FM interface. The largest exchange bias was achieved for OMO(5), having the thinnest outer shell, and by increasing the oxidation time, it almost had a decreasing trend compared to OMO(5). If the thickness of the AFM layer is so small, the anisotropy energy of the AFM layer would not be enough to pin the spins on the interface. However, as the thickness of the AFM shell increases, the anisotropy energy becomes sufficient to pin the spins. The variations in exchange bias with the enhancement of the AFM thickness can be explained through the role of AFM domains [5,40,76]. As domains are created in the outer AFM shell, more uncompensated spins become available at the interface. Subsequently, the exchange bias strengthens [5,40,76,79,80,81]. For the thicker AFM shell, the number of domains decreases. This is due to Malozemoff’s model, where it is pointed out that larger energy is necessary to create longer domain walls in thick AFM shells, which is not favorable, so the number of domains decreases. Thus, the uncompensated spins and the strength of the exchange bias decrease [5,40,76,79,80,81]. Accordingly, a decrease in the exchange bias value for thicker shell thickness than a critical size is expected. Moreover, interfaces’ quality and particle size considerably affect the exchange bias. In this case, although there is a distribution of particle sizes, longer oxidation times do not significantly affect the particle size and the interface quality (Figure 4). Increasing the percentage of Co_3_O_4_ by longer oxidation also could decrease the exchange bias. On the other hand, it is assumed that the core size becomes constant as the oxidation time increases. The variations in the thicknesses in the Co-oxide outer shell and the Co inner shell are tied to each other inversely. This means that the Co-oxide shell thickness changes at the expense of the simultaneous opposite change in the FM Co shell thickness. Hence, this dual effect should be considered to study the effect of increasing the oxidation time on the exchange bias. Sort et al. studied the exchange bias in the AFM/ FM/AFM thin film structure. Firstly, it was shown that there was a large difference in the squareness of the hysteresis loop and the exchange bias when the order of the AFM layer changed (AFM(7 nm)/FM and FM/AFM(7 nm)). This was ascribed to the structural difference, such as grain formation and interface roughness between the two configurations. Secondly, they revealed that the exchange bias in the AFM/FM/AFM structure is precisely the summation of the loop shifts of the two constituent AFM/FM and FM/AFM following the field cooling [5]. Therefore, although doubling the surface effects could increase the exchange bias, the loop shift direction and the amount of shift in each surface should also be considered in the summation. In our case, the thicknesses of both the FM shells and the AFM shells change during oxidation, and the exchange bias in the two interfaces changes simultaneously. The exchange bias has a declining trend following the increase in oxidation time. However, there is a slight increase or decrease in exchange bias values. The slight change could be due to the dual change in the thickness of the shells leading to nonmonotonic behavior in these samples. Vasilakaki et al. applied the Metropolis Monte Carlo method to study the effects of core size and shell thickness on the magnetic properties in an inverted structure. They showed that the exchange bias is directly or inversely proportional to the thickness of the FM layer, depending on the AFM core size. Hence, the variation in uncompensated spins at a different position in the core/shell was evaluated to unveil the reason for this feature. Any ferrimagnetic (ferromagnetic) shell alterations can change the uncompensated spins and alter the strength of the exchange bias. The surface spins affect the exchange bias for small core sizes the most. Meanwhile, the exchange coupling between the core and shell controls the exchange bias for the large core sizes. For the medium core size, both surface spins and interface are considered responsible for the exchange bias [27]. Therefore, all of these features with the dual effect in the core/shell/shell structure, which is a mixture of an inverted core/shell and a conventional core/shell, lead to a nonmonotonic variation in the exchange bias strength.

Figure 10c displays the change in vertical shifts with respect to oxidation time. The highest vertical shift was observed for a 5 min oxidation time, which decreased significantly as the oxidation time increased. This shift was partially due to spin-canting at the interface. As the spin canting decreased, the vertical shift also decreased [62]. Prolonged oxidation at high temperatures led to a decrease in vertical shift because of increased crystallinity and decreasing canted spins at the core–shell interface. Since exchange bias is related to uncompensated spins at the interface, this decrease in vertical shift aligns with the observed decline in exchange bias values with increasing oxidation time. The largest amount of vertical shift and exchange bias was seen in OMO(5). Furthermore, the variation in the vertical shift plot at 5 T suggests that with a larger external field, this shift might disappear. 

**Figure 11:**
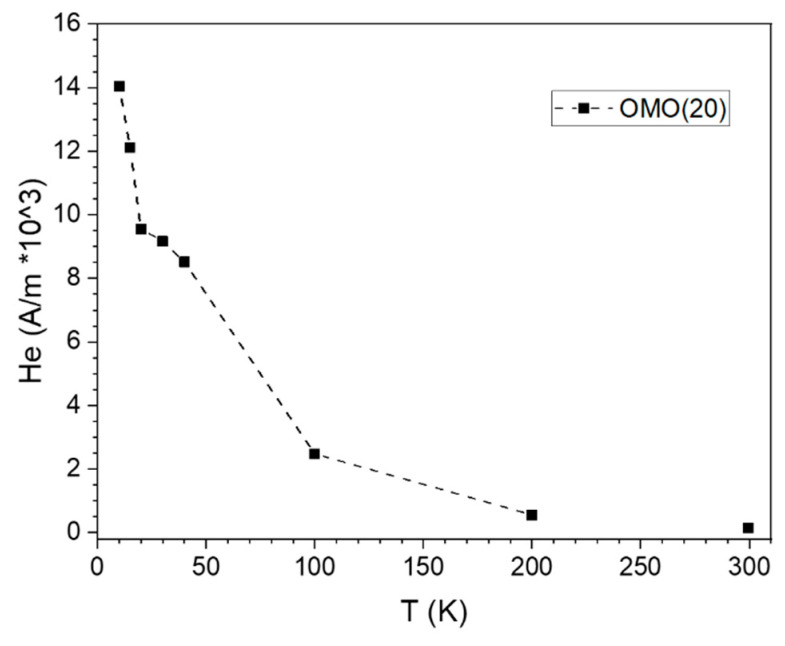
Exchange bias field of OMO(20) as a function of temperature, where the dotted line represents the general trend.

Figure 11 shows the temperature dependence of the exchange-bias field of the OMO(20) sample. As can be seen, the onset temperature of the exchange bias of OMO(20) starts beyond 200 K, and its value increases sharply at temperatures lower than 100 K. However, CoO is utilized considerably as an AFM material in the exchange-bias systems due to its high anisotropy and convenient Néel temperature (T_N_ = 291 K), which is very close to the room temperature. However, particle size strongly influences the transition temperature of this AFM (Néel and blocking temperatures). Hence, the particle size and the shell thickness variation affect the exchange bias at temperatures lower than the blocking temperature. Considering that the CoO nanoparticles have a lower Néel temperature than that of the bulk, the onset temperature of exchange bias is usually much lower than 290 K. These results reveal that the extra interface between FM and AFM layers in the Co-oxide/Co/Co-oxide core/shell provides the possibility to have an exchange bias close to the Néel temperature of CoO.

## 4. Conclusions

In conclusion, our study on the single inverted core/shell and core/shell/shell Co-oxide/Co and Co-oxide/Co//Co-oxide nanostructures has provided new insights into the enhancement of exchange bias and coercivity through the extra interface coupling formed at the shell–shell interface in the core/shell/shell structure. While it is true that similar increases in exchange bias and coercivity have been reported in thin film structures, to the best of our knowledge, this is the first report of such a significant increase in core/shell/shell structures that incorporate both inverted and normal structures.

One of the unique features of this structure is its mixture of inverted and normal structures, which highlights the significance of the combination of the two types of structures for the enhancement of magnetic properties. Our investigation of the effect of shell thickness on exchange bias revealed a non-monotonic behavior, which is typically observed in doubly inverted structures instead of single inverted ones. The exchange bias showed a declining trend with increasing shell thickness, reaching its highest value for the sample with the thinnest Co-oxide shell, contrary to our initial expectations. However, studies have shown that there is an upper limit to the AFM thickness beyond which exchange bias changes differently, with this limit varying for each sample. This declining behavior can be understood by considering the critical role of the antiferromagnetic domains and the energy required to form more of these domains with increasing shell thickness, as well as the increase in Co_3_O_4_ during the oxidation process. The simultaneous variation in Co shell and CoO shell thicknesses was also considered a reason for the non-monotonic behavior, representing a new result in the study of this type of structure.

Our results open up new possibilities for the design and synthesis of nanostructures with enhanced magnetic properties through the manipulation of interface coupling and shell thickness. The observation of the non-monotonic behavior of exchange bias and coercivity with shell thickness, in particular, highlights the importance of considering the interplay between different factors in the design of magnetic nanostructures. This study also provides a deeper understanding of the underlying mechanisms of exchange bias and coercivity enhancement in core/shell/shell structures and can serve as a guide for future research in this field. 

## Figures and Tables

**Table 1:** Summary of compositional and structural parameters extracted from magnetometry and XRD for the OMO-series. σ denotes saturation magnetization.

	Co % (σ/σbulk)	Co-Oxide % (1-σ/σbulk)	Co % (XRD)	Co-Oxide % (XRD)
OMO(0)	69.6	30.4	71.8	28.2
OMO(5)	39.7	60.3	36.6	63.4
OMO(20)	12.9	87.1	10.3	89.7
OMO(40)	10.4	89.6	2	98
OMO(60)	10.0	90	0	100
OMO(80)	9.6	90.4	0	100

## Data Availability

The data presented in this study are available on request from the corresponding author.
